# Maternal low thyroxin levels are associated with adverse pregnancy outcomes in a Chinese population

**DOI:** 10.1371/journal.pone.0178100

**Published:** 2017-05-23

**Authors:** Yong Zhang, Xiaobei Dai, Shuai Yang, Chen Zhang, Mi Han, He-Feng Huang, Jianxia Fan

**Affiliations:** 1 International Peace Maternity and Child Health Hospital, School of Medicine, Shanghai Jiao Tong University, Shanghai, China; 2 Institute of Embryo-Fetal Original Adult Disease Affiliated with Shanghai Jiao Tong University School of Medicine, Shanghai, China; Baylor College of Medicine, UNITED STATES

## Abstract

Although thyroid dysfunction in early pregnancy may have adverse effects on pregnancy outcomes, few studies have examined the relationship between maternal low free thyroxin (FT4) levels in both first and third trimesters of pregnancy and the incidence of adverse pregnancy outcomes. We hypothesized that low FT4 levels in either first or third trimesters of pregnancy may have different effects on pregnancy outcomes. The study included 6,031 mothers who provided both first and third pregnancy serum samples for analyses of thyroid function. Adverse pregnancy outcomes, such as gestational diabetes mellitus (GDM), pregnancy-induced hypertension and preeclampsia, were diagnosed using the oral glucose tolerance test, blood pressure and urine protein test. Serum metabolites like adenosine and its analogues were identified using hydrophilic interaction liquid chromatography (HILIC)-tandem mass spectrometry (MS/MS). The incidence of hypothyroidism in pregnant women tended to increase with age and pre-pregnancy body mass index (BMI). The incidence of GDM was negatively correlated with maternal FT4 levels during early pregnancy while the incidence of preeclampsia was negatively correlated with maternal FT4 levels during late pregnancy. The incidence of pregnancy-induced hypertension was not significantly correlated with maternal FT4 levels. The women who had isolated maternal hypothyroxemia (IMH) in the third trimester of pregnancy had an increased risk of developing preeclampsia. Some metabolites like adenosine and its analogues in the serum were significantly changed in pregnant mothers with IMH. In conclusion, low FT4 levels during pregnancy are a risk factor for GDM and preeclampsia. Adenosine and its analogues may be important bridges between IMH and preeclampsia.

## Introduction

During pregnancy, maternal thyroid hormone levels exhibit large changes due to the influence of varying hormone levels, and thyroid dysfunction is related to many adverse outcomes in both mothers and fetuses, such as preterm delivery, intrauterine growth restriction, preeclampsia and lower birth weight [1–3]. Gestational hypothyroxemia refers to a lower serum free thyroxin (FT4) level that is less than 10% of the reference with the normal thyroid-stimulating hormone (TSH) level [4]. Isolated maternal hypothyroxemia (IMH) is the thyroid peroxidase antibody (TPOAb)-negative type of hypothyroxemia. In our population, the prevalence of maternal thyroid dysfunction is 15.0%. Given the prevalence and adverse outcomes related to maternal thyroid dysfunction, many studies which focused on improving pregnancy outcomes by screening pregnant women for thyroid disease have been conducted in the past few years [5–8]. Our previous study demonstrated that low thyroid hormone levels in early pregnancy are a risk factor for gestational diabetes mellitus (GDM) [9]. However, in most of the IMH studies, the FT4 levels were measured before 20 weeks of pregnancy and were not measured in the later trimesters of pregnancy [5, 10–13]. Maternal FT4 levels must be monitored in both the first and third trimesters of pregnancy, and its relationship with adverse pregnancy outcomes must be evaluated. In the present study, we aimed to explore the correlation between the FT4 levels in the first trimester or third trimester of pregnancy and adverse pregnancy outcomes.

Because thyroxin affects important metabolic pathways such as AMPK (Adenosine 5’-monophosphate (AMP)-activated protein kinase) pathway and PI3K/Akt pathway, altered levels of metabolites in patients with hypothyroxemia have been the focus of many thyroid disease studies [14–16]. In recent years, metabolomic studies have provided the advanced methods necessary to identify changing metabolite levels, resulting in rapid progress in disease biomarker discovery [17–19]. A metabolomic analysis using serum samples from patients with hypothyroxemia has not been reported. We hypothesized that metabolites such as adenosine or its analogues which were involved in AMPK signaling pathway may be altered in patients with hypothyroxemia. In the present study, we identified the serum metabolites by metabolomics analysis in patients with hypothyroxemia to address the potential relationship between gestational hypothyroxemia and adverse pregnancy outcomes.

## Methods

### Ethics

This study was performed in accordance with the relevant guidelines and regulations. The project was approved by the Ethics Committee of the International Peace Maternity and Child Health Hospital, School of Medicine, Shanghai Jiaotong University. Written informed consent was obtained from each subject before samples were collected. The data analysis procedures conformed to the principles of the Declaration of Helsinki.

### Patient enrollment

The study group consisted of pregnant women undergoing their first and third trimesters prenatal screenings at the International Peace Maternity and Child Health Hospital. According to the recommendations of the National Academy of Clinical Biochemistry (NACB) for tests determining thyroid function (TFT), the inclusion criteria were: single birth, Han Chinese women, no history of thyropathy or autoimmune disease, no goiters, thyroid peroxidase antibody (TPOAb)-negative, no use of medicines affecting the thyroid hormone levels, and TSH levels that were within the reference intervals during first and third trimesters of pregnancy.

From January 2013 to May 2014, a total of 6,031 pregnant women (aged 21–43 years old) were enrolled in the study. We collected the subjects’ ages, body mass indexes (BMIs), and TSH and FT4 levels during the 9^th^-12^th^ weeks of pregnancy (first trimester) and 32^nd^-36^th^ weeks of pregnancy (third trimester). Blood was collected from eligible pregnant women who consented to enroll in the study upon their visit to the antenatal clinic. Authors had access to information that could identify individual participants during or after data collection.

Fasting blood samples were drawn from the median cubital vein, and the serum was separated by centrifugation within 6 h. TSH, FT4 and TPOAb levels were measured with Abbott (ARCHITECT i2000; Abbott, Chicago, USA) kits according to the manufacturer’s protocol.

Diagnosis for GDM: fasting blood glucose level greater than 5.1 mmol/l or 1 and 2 h blood glucose levels after the oral glucose tolerance test (OGTT) greater than 10.0 mmol/l and 8.5 mmol/l, respectively. OGTT was conducted within 24^th^-28^th^ weeks of pregnancy.

Diagnosis for gestational hypertension: systolic blood pressure ≥140 mmHg or diastolic blood pressure ≥90 mmHg during pregnancy, which returned to normal within 12 weeks after delivery with negative urine proteins according to ACOG guidelines of 2013.

Diagnosis for preeclampsia: systolic blood pressure ≥140 mmHg or diastolic blood pressure ≥90 mmHg after 20 weeks of pregnancy, with normal blood pressure before 20 weeks of pregnancy, with urine protein levels ≥0.3 g/24 h or random urine protein positive, or with the syndromes according to ACOG definitions of 2013.

### Metabolomic analysis

The blood samples from normal pregnant women and patients with IMH controlled for age, BMI and TSH were collected for metabolomics analysis. The metabolites were extracted, and the metabolomic analysis was conducted at Applied Protein Technology (APT), Shanghai, China. Briefly, 50 μl of serum was added to 450 μl of methanol containing internal standards to inactivate the native enzymes. The extract solution was thoroughly mixed with 500 μl of chloroform and 200 μl of Milli-Q water and centrifuged at 12,000 *g* for 15 min. The 400 μl upper aqueous layer was centrifugally filtered through a Millipore cutoff filter to remove the protein content. The filtrate was then centrifugally concentrated and re-suspended in Milli-Q water for the metabolomic analysis at APT via hydrophilic interaction liquid chromatography coupled with tandem mass-spectrometry (HILIC-MS/MS) using previously described methods [20, 21].

### Statistical analysis

All statistical analyses were performed using SPSS 19.0 software. Levene’s test was used determine the homogeneity of the variances; Kolmogorov-Smirnov (D test) was used to assess the normal distribution of the sample. The U test was used to compare the FT4 levels in patients with various pregnancy outcomes. Pearson’s test was used to analyze the correlation between the FT4 and TSH levels, and a logistic regression analysis was used to determine the relationship between the FT4 levels and pregnancy outcomes. Student’s *t* test was used to compare the serum metabolite levels. Differences were considered statistically significant at *P*<0.05.

## Results

### Comparisons of the clinical data from four groups of pregnant women with different FT4 trends during pregnancy

Specific reference ranges of FT4 during first and third trimesters of pregnancy were shown in Table 1, and 10^th^ percentile (P10) was the cutoff value of FT4 according to the guidelines of National Academy of Clinical Biochemistry (NACB). We divided the FT4 levels into two groups: a low FT4 level and a normal FT4 level. After combining these groups with the first trimester and third trimester of pregnancy, we divided the pregnant women into 4 groups: normal/normal group: early normal/late normal (n = 4672), low/low group: early low/late low (n = 228), low/normal group: early low/late normal (n = 608) and normal/low group: early normal/late low (n = 523). Women in the low/low group were older than the women in the normal/normal group, and the average BMI in the low/low group was higher than the normal/normal group. The average TSH level in the first trimester was higher in women in the low/low group than in women in the normal/normal group. Women in the low/normal group also were older and had a higher BMI and TSH level in the first trimester than the women in the normal/normal group. Women in the normal/low group had a higher BMI and TSH level in the third trimester than the women in the normal/normal group (Table 2). Besides, comparisons of the clinical data from normal pregnant women and patients with adverse pregnancy outcomes were shown in Table 3.

**Table 1:** Specific reference ranges of FT4 during first and third trimester of pregnancy.

	First Trimester					Third Trimester				
	P2.5	P5	P10	P50	P97.5	P2.5	P5	P10	P50	P97.5
FT4 (pmol/L)	12.8	13.1	13.5	15.3	18.6	9.6	10.0	10.4	11.8	14.3

FT4: free T4; P2.5: 2.5th Percentile; P5: 5th Percentile; P10: 10th Percentile; P50: 50th Percentile; P97.5: 97.5th Percentile.

**Table 2:** Comparisons of the clinical data from four groups of pregnant women with different FT4 trends during pregnancy.

Characteristics	Normal/normal	Low/low	Low/normal	Normal/low
	n = 4672	n = 228	n = 608	n = 523
Age (year)	29.9±3.1	30.8±3.2 **	31.0±3.3 **	30.2±3.1
BMI (kg/m^2^)	21.83±2.80	23.59±2.95 **	23.48±5.66 **	22.25±2.75 **
Gestational weeks (at Delivery)	38.9±1.7	39.0±1.2	38.9±1.6	39.0±1.5
TSH (mIU/L)				
First Trimester	1.16±0.64	1.37±0.58 **	1.33±0.61 **	1.21±0.66
Third Trimester	1.53±0.67	1.61±0.65	1.59±0.66	1.61±0.67 *
FT4 (pmol/L)				
First Trimester	15.45±1.18	12.61±0.65 **	12.84±0.54 **	15.02±1.11
Third Trimester	12.11±0.98	9.60±0.59 **	11.65±0.85	9.81±0.45 **

BMI: Body mass index; TSH: Thyroid-stimulating hormone;

*P<0.05,

**P<0.01, compared with Normal/normal group.

**Table 3:** Comparisons of the clinical data from normal pregnant women and patients with adverse pregnancy outcomes.

Characteristics	Normal	GDM	Preeclampsia	Gestational hypertension
	n = 5175	n = 533	n = 99	n = 224
Age (year)	29.93±3.08	31.20±3.61 **	30.01±3.26	30.40±3.29 *
BMI (kg/m^2^)	21.79±3.12	23.42±3.30 **	23.65±3.39 **	24.57±3.80 **
Body weight (kg)	57.90±0.11	61.91±0.42 **	62.21±1.04 **	65.93±0.80 **
Gestational weeks (at Delivery)	39.05±1.32	38.61±1.21 **	38.76±1.20 *	38.32±1.71 **
TSH (mIU/L)				
First Trimester	1.19±0.64	1.18±0.65	1.18±0.62	1.22±0.64
Third Trimester	1.54±0.66	1.54±0.70	1.71±0.74 *	1.58±0.69
FT4 (pmol/L)				
First Trimester	15.09±1.44	14.73±1.43 **	14.78±1.57 *	14.85±1.39 *
Third Trimester	11.78±1.20	11.78±1.12	11.41±1.50 **	11.59±1.23 *

BMI: Body mass index; TSH: Thyroid-stimulating hormone;

*P<0.05,

**P<0.01, compared with normal group.

### Relationship between the FT4 levels and adverse outcomes in first and third trimesters of pregnancy

**Fig 1:**
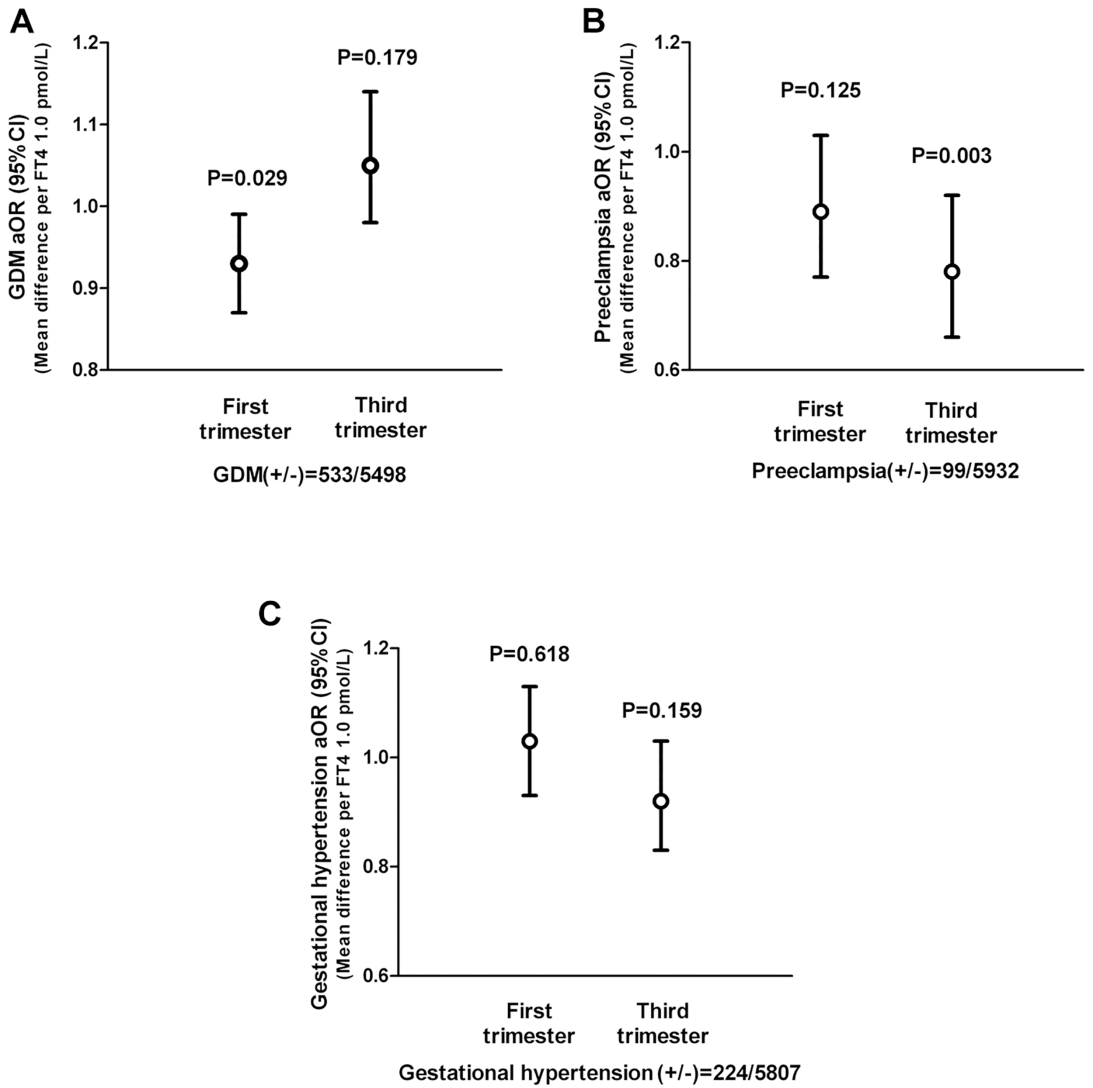
Relationship between the FT4 levels and adverse outcomes in first and third trimesters of pregnancy. A. The relationship between FT4 and GDM. B. The relationship between FT4 and preeclampsia. C. The relationship between FT4 and gestational hypertension. GDM: Gestational diabetes mellitus; FT4: Free T4; aOR: Adjusted odds ratio; CI: confidence interval.

We adjusted for the confounding factors of age, BMI and TSH levels by logistic regression model to calculate the odds ratio and found that the first trimester FT4 levels were negatively correlated with the occurrence of GDM (β = -0.074, *P* = 0.029), but the third trimester FT4 levels were not correlated with the occurrence of GDM (β = 0.052, *P* = 0.179) (Fig 1A). Third trimester FT4 levels were negatively correlated with the occurrence of preeclampsia (β = -0.252, *P* = 0.003), but first trimester FT4 levels were not correlated with the occurrence of preeclampsia (β = -0.115, *P* = 0.125) (Fig 1B). However, neither first trimester FT4 levels nor third trimester FT4 levels were correlated with the occurrence of gestational hypertension (*P* = 0.618 for first trimester, *P* = 0.159 for third trimester) (Fig 1C).

### Correlation between the maternal FT4 trends and GDM in pregnancy

Women in the low/normal group had a 1.61-fold higher risk of developing GDM than the women in the normal/normal group (confidence interval (CI): 1.25–2.09), and the women in the low/low and normal/low groups did not show significant differences in the risk of developing GDM compared with that of the women in the normal/normal group. However, after adjusting for confounding factors such as age, BMI and TSH levels, women in the low/low, low/normal or normal/low groups did not show dramatic differences in their risk of developing GDM from the women in the normal/normal group (Table 4).

**Table 4:** Correlation between the maternal FT4 trends and GDM in pregnancy.

FT4 group	n (%)	OR (95% CI)	aOR (95% CI) ^b^
Normal/normal	396 (9.26)	Ref	Ref
Low/low	22 (10.68)	1.15 (0.73–1.81)	0.86 (0.54–1.37)
Low/normal	79 (14.93)	1.61 (1.25–2.09) *	1.18 (0.90–1.56)
Normal/low	36 (7.39)	0.80 (0.56–1.14)	0.75 (0.52–1.07)

GDM: Gestational diabetes mellitus; FT4: Free T4; aOR: Adjusted odds ratio; CI: confidence interval;

^b.^ Adjusted age, BMI and TSH.

* *P*<0.05 compared with Normal/normal group.

### Correlation between the maternal FT4 trends and preeclampsia in pregnancy

As shown in Table 5, the women in the low/low group had a 2.66-fold higher risk of developing preeclampsia than the women in the normal/normal group (CI: 1.26–5.62). In addition, the women in the normal/low group had a 2.16-fold higher risk of developing preeclampsia than the women in the normal/normal group (CI: 1.22–3.82). After adjusting for confounding factors such as age, BMI and TSH levels, the women in the low/low and normal/low groups still had a 2.62- and 2.18-fold higher risk of developing preeclampsia, respectively, than the women in the normal/normal group. The women in the low/normal group did not show significant differences in the risk of developing preeclampsia from the women in the normal/normal group (*P* = 0.405).

**Table 5:** Correlation between the maternal FT4 trends and preeclampsia in pregnancy.

FT4 group	n (%)	OR (95% CI)	aOR (95% CI) ^b^
Normal/normal	63 (1.35)	Ref	Ref
Low/low	8 (3.51)	2.66 (1.26–5.62) *	2.62 (1.23–5.62) *
Low/normal	13 (2.14)	1.60 (0.87–2.92)	1.33 (0.68–2.59)
Normal/low	15 (2.87)	2.16 (1.22–3.82) *	2.18 (1.22–3.87) *

FT4: Free T4; aOR: Adjusted odds ratio; CI: confidence interval;

^b.^ Adjusted age, BMI and TSH.

* *P*<0.05 compared with Normal/normal group.

### Comparisons of the blood metabolite levels between the groups with normal FT4 levels and low FT4 levels during pregnancy

**Fig 2:**
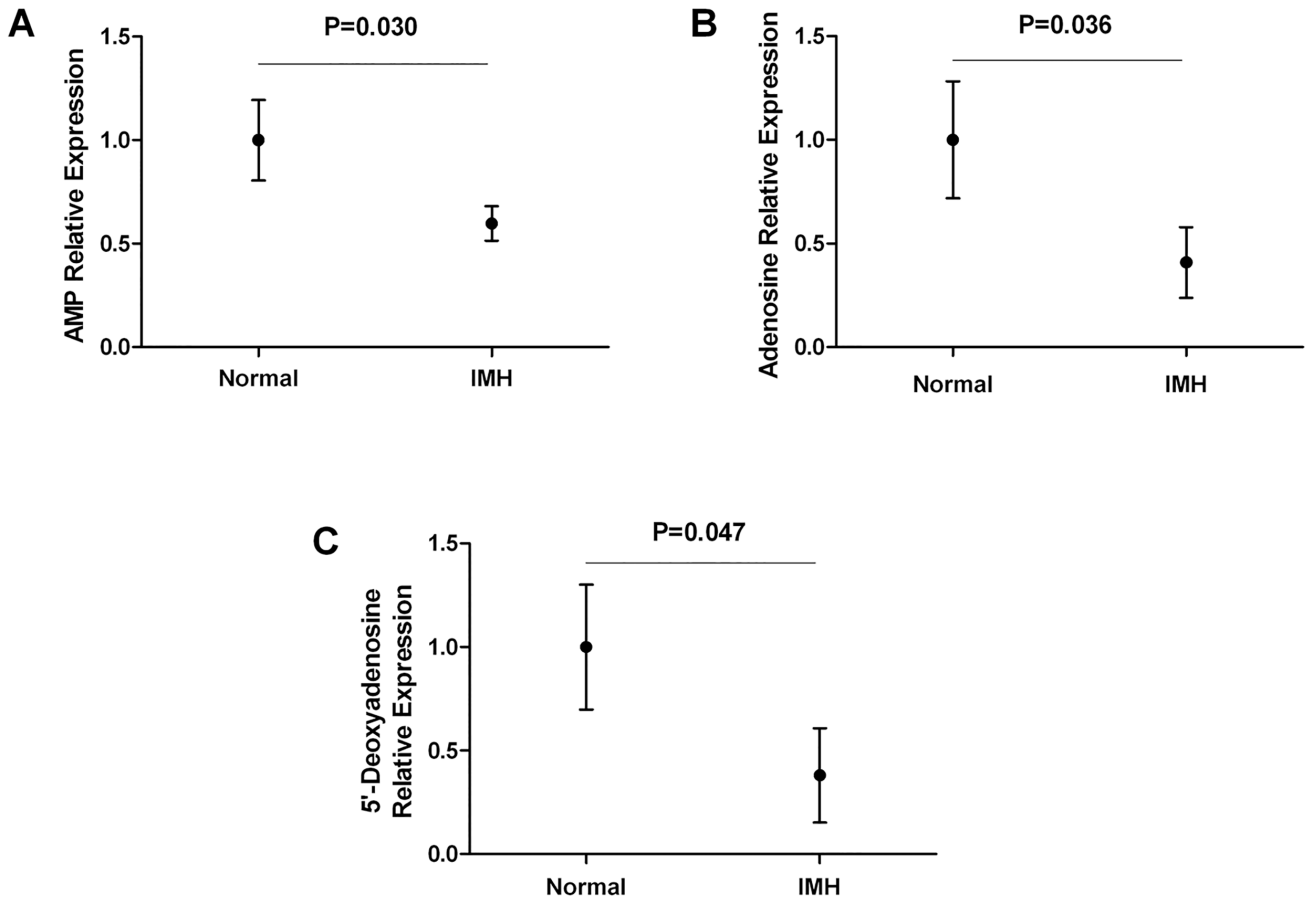
Comparisons of the blood metabolite levels between the groups with normal FT4 levels and low FT4 levels during pregnancy. Comparisons of adenosine monophosphate (AMP), adenosine and 5’-deoxyadenosine between the groups with normal FT4 (Normal) and low FT4 (IMH) during the third trimester of pregnancy.

In the present study, we used HILIC-MS/MS to obtain a comprehensive IMH metabolic profile. One hundred ten metabolites were obtained from the serum of 3 patients with IMH and preeclampsia and 3 healthy control subjects during the third trimester of pregnancy. The targeted metabolomics assay had internal quality control samples to evaluate the stability and reproducibility of the results. Of these metabolites, the levels of 8 metabolites (adenosine, adenosine monophosphate, 5’-deoxyadenosine, aminohippuric acid, glycocholic acid, D-glucuronic acid, allantoic acid, sepiapterin) were down-regulated and the levels of 2 metabolites (L-homocysteine, hordenine) were up-regulated in patients with IMH compared with those of healthy controls during pregnancy (S1 Table). The levels of adenosine, adenosine monophosphate (AMP) and 5’-deoxyadenosine were decreased in patients with IMH during the third trimester of pregnancy compared with those in the healthy control subjects (Fig 2). It is possible that adenosine and its analogues or its related kinases may be important bridges between IMH and preeclampsia.

## Discussion

In the present study, the risk of developing GDM increased when the maternal FT4 level decreased. Gestational hypothyroxemia and GDM are both specific endocrine diseases that harm both mothers and babies. In a previous study, women who developed GDM showed a mean FT4 level that was significantly lower than the level observed in healthy pregnant women and women with diabetes type 1 [22]. In addition, pregnant women with IMH have a higher risk of developing insulin resistance and GDM [6, 23]. In fact, a reduced FT4 level in the maternal circulation is associated with an increase in the occurrence of GDM and impaired development of the central nervous system in children from pregnancies affected by these diseases [24–26]. Our present study found that the incidence of GDM was correlated with low FT4 levels in first trimester of pregnancy, but not in third trimester of pregnancy. This may due to the chronic effects of thyroxin on the target organs to induce glucose intolerance and insulin resistance. GDM is associated with increased synthesis and release of vasodilators such as nitric oxide (NO) in the endothelium [27, 28]. Moreover, thyroid hormones are also involved in NO synthesis and release [29, 30], but the potential contribution of the reduced circulating FT4 levels to the deregulation of endothelial function observed in GDM is unclear and must be clarified in future research [31].

In the current study, we also investigated the associations between IMH and the risk of developing hypertension during pregnancy. IMH was not associated with gestational hypertension. Hypothyroidism has been shown to have various vascular effects, including endothelial cell dysfunction [32]. Therefore, multiple studies have investigated the association between thyroid dysfunction and hypertensive disorders during pregnancy [6, 11, 26, 33, 34]. In some of these studies, mothers with hypothyroidism were reported to have an increased risk of developing hypertensive disorders [33, 34], whereas other studies did not identify any associations [6, 11, 26]. The differences between these studies might be because not all studies controlled for the potentially confounding factors, such as age, BMI, smoking, thyroid autoimmunity, ethnicity and socioeconomic status. As reported in a recent study, hypothyroidism and hypothyroxemia were not associated with hypertensive disorders, based on data obtained from the early trimester of pregnancy [7]. Our present study also considered the late trimester and did not identify an association between IMH and gestational hypertension. Based on these results, there is no obvious correlation between IMH and gestational hypertension in both the first and third trimesters of pregnancy.

Our study shows, for the first time, that IMH detected in the third trimester of pregnancy is strongly correlated with preeclampsia in a large Chinese population. To our knowledge, this study is the first large investigation in which long-term monitoring of the FT4 levels in Chinese mothers with hypothyroxemia during pregnancy has been performed to evaluate the risk of developing preeclampsia. In previous studies, low maternal FT4 levels in early pregnancy did not correlate with preeclampsia [35], but the correlation of the FT4 trends during the third trimester of pregnancy with preeclampsia has not been clarified. In the present study, low FT4 levels in the third trimester of pregnancy increased the risk of developing preeclampsia. Hence, the identification of the changes in the thyroid hormone levels during pregnancy might be helpful in preventing the occurrence of preeclampsia. Our previous report has shown that universal screening for FT4 levels in pregnant women could effectively reduce the misdiagnosis rate of thyroid dysfunction [36]. Together, thyroid dysfunction during first and third trimesters of pregnancy may correlate with different adverse pregnancy outcomes.

In the current study, we explored and identified significant differences in the serum metabolite levels in pregnant mothers with IMH compared those in with healthy controls excluding the confounding effects of BMI and age. Using HILIC-MS/MS, we detected 110 known metabolites, among which the levels of 8 metabolites decreased and 2 metabolites increased in pregnant mothers with IMH compared to those in the healthy controls. Interestingly, the adenosine, adenosine monophosphate and 5’-deoxyadenosine levels were significantly reduced in pregnant mothers with IMH, indicating that adenosine and its analogues or kinases may be affected by thyroxin. It is well documented that adenosine is a vasodilator in the placenta, coronary, cerebral and muscular circulation, in several conditions including hypoxia and exercise. Hence, adenosine and its analogues or related kinases may be involved in both diabetes and hypertension conditions [37–39]. Besides, thyroid hormone may activate liver and muscle adenosine 5’-monophosphate activated protein kinase to regulate mitochondrial fatty acid oxidation and energy metabolism [40, 41]. The metabolite study was performed in normal pregnant women and IMH patients with preeclampsia in third trimester of pregnancy and we concluded that the differential expressed metabolites such as adenosine might contribute to preeclampsia in IMH patients. Adenosine and its analogues or related kinases may be important bridges between hypothyroxemia and preeclampsia. The exact roles of adenosine and its analogues in the relationship between hypothyroxemia and adverse pregnancy outcomes need to be explored in future studies.

There are still some limitations in this study. All of the participants in our study were TPOAb negative, and we did not examine the relationship between TPOAb positive and adverse pregnancy outcomes, although some research has shown that TPOAb positive in early pregnancy is associated with an increased risk of GDM and preeclampsia [42, 43]. Another limit in this study is the lack of mechanism study for the metabolites role in hypothyroxemia and preeclampsia. Further investigation should be done to reveal the significance of TPOAb and metabolites between thyroxin levels in different trimesters of pregnancy and adverse pregnancy outcomes.

In conclusion, the maternal free thyroxin levels during pregnancy were correlated with the risk of developing GDM and preeclampsia in the present study. Maternal low free thyroxin levels during first and third trimesters of pregnancy may correlate with the occurrence of different adverse pregnancy outcomes.

## Supporting information

S1 Table List of metabolites identified by HILIC-MS/MS. One hundred ten metabolites were detected. Of these metabolites, the levels of 8 metabolites (adenosine, adenosine monophosphate, 5’-deoxyadenosine, aminohippuric acid, glycocholic acid, D-glucuronic acid, allantoic acid, sepiapterin) were decreased while the levels of 2 metabolites (L-homocysteine, hordenine) were increased in patients with IMH compared with healthy controls (Con) during pregnancy. * *P*<0.05 vs. Con.
(DOCX)

S2 Table STROBE Statement—Checklist of items that should be included in reports of observational studies. (DOCX)
