# Supplementary material for: Maternal low thyroxin levels are associated with adverse pregnancy outcomes in a Chinese population
Source: PLoS One. 2017 May 23;12(5):e0178100. doi: 10.1371/journal.pone.0178100 (PMC5441606; doi:10.1371/journal.pone.0178100)
Supplement: S1 Table — One hundred ten metabolites were detected. Of these metabolites, the levels of 8 metabolites (adenosine, adenosine monophosphate, 5’-deoxyadenosine, aminohippuric acid, glycocholic acid, D-glucuronic acid, allantoic acid, sepiapterin) were decreased while the levels of 2 metabolites (L-homocysteine, hordenine) were increased in patients with IMH compared with healthy controls (Con) during pregnancy. * P<0.05 vs. Con. (DOCX) [file pone.0178100.s001.docx]

**S1 Table. List of metabolites identified by HILIC-MS/MS.**

| **Metabolite name** | **Con** | | **IMH** | | **IMH vs. Con** | |
| --- | --- | --- | --- | --- | --- | --- |
|  | **Mean** | **SE** | **Mean** | **SE** | **Ratio** | **p value** |
| Adenosine monophosphate (AMP) | 1.1E+04 | 1.3E+03 | 6.7E+03 | 5.4E+02 | 0.598 | 0.030* |
| Adenosine | 1.6E+06 | 2.5E+05 | 6.4E+05 | 1.5E+05 | 0.408 | 0.036* |
| 5'-Deoxyadenosine | 7.0E+03 | 1.2E+03 | 2.7E+03 | 9.1E+02 | 0.381 | 0.047* |
| Aminohippuric acid | 7.4E+02 | 3.1E+01 | 8.4E+01 | 1.8E+01 | 0.113 | 0.000* |
| Glycocholic acid | 4.0E+04 | 9.2E+03 | 9.4E+03 | 9.3E+02 | 0.237 | 0.031* |
| D-Glucuronic acid | 3.1E+04 | 9.9E+02 | 2.5E+04 | 1.5E+03 | 0.819 | 0.037* |
| Allantoic acid | 1.3E+03 | 2.3E+02 | 5.7E+02 | 3.3E+01 | 0.450 | 0.038* |
| Sepiapterin | 8.6E+03 | 3.0E+02 | 7.1E+03 | 4.0E+02 | 0.826 | 0.040* |
| L-Homocysteine | 1.3E+03 | 3.3E+02 | 2.8E+03 | 3.5E+02 | 2.095 | 0.040* |
| Hordenine | 2.5E+03 | 5.4E+01 | 3.1E+03 | 2.1E+02 | 1.258 | 0.042* |
| sn-Glycerol 3-phosphate | 2.4E+03 | 2.1E+02 | 1.6E+03 | 2.4E+02 | 0.652 | 0.057 |
| Pseudouridine | 4.7E+04 | 5.9E+02 | 7.0E+04 | 8.6E+03 | 1.478 | 0.059 |
| N4-Acetylcytidine | 1.3E+05 | 1.3E+04 | 1.8E+05 | 1.3E+04 | 1.370 | 0.059 |
| L-Methionine | 1.4E+06 | 6.5E+04 | 1.1E+06 | 1.0E+05 | 0.794 | 0.071 |
| Cytidine diphosphate choline (CDPcholine) | 3.3E+02 | 1.4E+02 | 6.9E+02 | 5.5E+01 | 2.112 | 0.075 |
| Glyceric acid | 3.1E+03 | 4.7E+02 | 2.0E+03 | 1.8E+02 | 0.623 | 0.079 |
| N-Acetyl-L-tyrosine | 1.1E+03 | 1.4E+02 | 3.2E+03 | 9.0E+02 | 2.984 | 0.083 |
| Flavone | 3.3E+04 | 6.3E+03 | 4.8E+04 | 7.5E+02 | 1.442 | 0.084 |
| L-Tryptophan | 4.2E+06 | 2.6E+05 | 3.6E+06 | 9.9E+04 | 0.856 | 0.088 |
| 2-Thiocytidine | 1.8E+04 | 6.0E+03 | 5.6E+03 | 1.4E+03 | 0.306 | 0.107 |
| Riboflavin (Vitamin B2) | 8.3E+02 | 8.2E+01 | 1.8E+03 | 4.8E+02 | 2.194 | 0.112 |
| beta-D-Glucosamine | 2.2E+03 | 5.3E+02 | 9.9E+02 | 2.8E+02 | 0.451 | 0.116 |
| Hypoxanthine | 2.0E+06 | 1.2E+05 | 3.9E+06 | 9.9E+05 | 1.989 | 0.123 |
| Acetyl-DL-Leucine | 2.9E+03 | 1.5E+02 | 6.5E+03 | 1.9E+03 | 2.266 | 0.127 |
| L-2-Hydroxygluterate | 1.3E+03 | 5.3E+01 | 1.6E+03 | 1.3E+02 | 1.197 | 0.128 |
| L-Leucine | 6.3E+06 | 5.8E+05 | 4.6E+06 | 6.6E+05 | 0.737 | 0.135 |
| L-Serine | 2.5E+05 | 4.1E+03 | 2.2E+05 | 1.7E+04 | 0.875 | 0.150 |
| Dimethylglycine | 6.6E+05 | 7.2E+04 | 9.7E+05 | 1.6E+05 | 1.469 | 0.153 |
| L-Arginine | 2.3E+07 | 2.5E+06 | 1.6E+07 | 3.2E+06 | 0.697 | 0.160 |
| Thymine | 1.5E+03 | 9.9E+01 | 7.0E+02 | 4.3E+02 | 0.479 | 0.162 |
| L-2-Aminoadipic acid | 1.4E+05 | 3.6E+04 | 7.4E+04 | 9.1E+03 | 0.537 | 0.163 |
| L-Phenylalanine | 1.9E+07 | 2.4E+06 | 1.4E+07 | 1.6E+06 | 0.744 | 0.176 |
| beta-Hydroxybutyric acid | 7.5E+03 | 2.3E+03 | 3.6E+03 | 5.7E+02 | 0.486 | 0.180 |
| 5-Aminolevulinic acid | 2.0E+03 | 1.4E+02 | 1.5E+03 | 2.6E+02 | 0.769 | 0.190 |
| Guanosine | 4.8E+04 | 7.4E+03 | 3.6E+04 | 4.7E+03 | 0.744 | 0.227 |
| Urocanic acid | 1.1E+05 | 1.3E+04 | 1.8E+05 | 5.1E+04 | 1.676 | 0.232 |
| 6-Hydroxynicotinic acid | 9.3E+02 | 4.8E+01 | 7.2E+02 | 1.4E+02 | 0.779 | 0.235 |
| Kynurenic acid | 5.4E+04 | 5.4E+03 | 4.5E+04 | 3.2E+03 | 0.841 | 0.249 |
| L-Asparagine | 4.5E+05 | 1.0E+05 | 3.0E+05 | 3.6E+04 | 0.673 | 0.249 |
| Glycerophosphocholine | 6.2E+06 | 1.9E+05 | 8.1E+06 | 1.4E+06 | 1.311 | 0.257 |
| L-Valine | 1.2E+07 | 1.3E+06 | 9.8E+06 | 1.2E+06 | 0.809 | 0.257 |
| L-Methionine sulfoxide | 1.2E+05 | 1.8E+04 | 1.9E+05 | 5.1E+04 | 1.610 | 0.261 |
| 4-Guanidinobutyric acid | 4.5E+04 | 3.7E+03 | 3.3E+04 | 9.4E+03 | 0.726 | 0.288 |
| Uric acid | 4.5E+04 | 4.1E+03 | 5.8E+04 | 9.9E+03 | 1.280 | 0.303 |
| N1-Methyl-2-pyridone-5-carboxamide | 9.1E+05 | 2.6E+05 | 6.0E+05 | 1.1E+05 | 0.661 | 0.337 |
| N-Acetyl-L-phenylalanine | 9.1E+03 | 3.8E+03 | 1.9E+04 | 8.3E+03 | 2.096 | 0.337 |
| N-Formylmethionine | 1.4E+03 | 3.9E+02 | 2.6E+03 | 9.7E+02 | 1.793 | 0.341 |
| Pantothenic acid | 4.0E+04 | 9.4E+03 | 5.1E+04 | 5.4E+03 | 1.288 | 0.352 |
| Glycine | 9.9E+04 | 8.7E+03 | 8.8E+04 | 6.6E+03 | 0.885 | 0.352 |
| Ureidopropionic acid | 4.1E+03 | 1.0E+03 | 5.4E+03 | 5.9E+02 | 1.294 | 0.357 |
| Imidazole | 1.5E+04 | 1.2E+03 | 2.0E+04 | 4.6E+03 | 1.319 | 0.360 |
| 5-Aminopentanoic acid | 3.4E+04 | 5.2E+03 | 2.8E+04 | 2.4E+03 | 0.827 | 0.362 |
| 5'-Methylthioadenosine | 3.9E+04 | 2.1E+03 | 4.7E+04 | 6.7E+03 | 1.181 | 0.363 |
| gamma-Glutamyl-L-methionine | 2.6E+03 | 3.7E+02 | 2.2E+03 | 1.6E+02 | 0.842 | 0.365 |
| N-Acetylaspartylglutamic acid (NAAG) | 1.2E+03 | 1.3E+02 | 1.0E+03 | 9.0E+01 | 0.866 | 0.369 |
| N-Acetylglutamine | 5.8E+03 | 1.7E+03 | 1.0E+04 | 4.0E+03 | 1.733 | 0.382 |
| Nicotinamide | 1.4E+05 | 4.8E+04 | 2.0E+05 | 4.0E+04 | 1.446 | 0.389 |
| gamma-L-Glutamyl-L-valine | 6.8E+03 | 8.4E+02 | 5.7E+03 | 9.1E+02 | 0.826 | 0.391 |
| S-Adenosylhomocysteine | 7.7E+03 | 1.0E+03 | 9.0E+03 | 1.0E+03 | 1.177 | 0.410 |
| Adenosine 3',5'-cyclic monophosphate (cAMP) | 1.7E+03 | 2.8E+02 | 1.3E+03 | 3.7E+02 | 0.757 | 0.415 |
| L-Glutamic acid | 2.9E+06 | 2.8E+05 | 3.7E+06 | 8.5E+05 | 1.282 | 0.418 |
| Alpha-N-Phenylacetyl-L-glutamine | 3.9E+05 | 2.1E+05 | 6.6E+05 | 2.3E+05 | 1.706 | 0.425 |
| L-Lactic acid | 5.1E+05 | 3.0E+04 | 5.6E+05 | 5.1E+04 | 1.102 | 0.426 |
| 1-Methylxanthine | 5.2E+03 | 2.4E+03 | 3.1E+03 | 8.1E+02 | 0.596 | 0.451 |
| taurine | 1.1E+06 | 1.6E+04 | 1.2E+06 | 1.2E+05 | 1.086 | 0.471 |
| Inosine | 3.6E+05 | 4.7E+04 | 3.2E+05 | 3.3E+04 | 0.879 | 0.485 |
| N-Acetylputrescine | 2.0E+05 | 4.2E+04 | 2.5E+05 | 4.9E+04 | 1.245 | 0.496 |
| gamma-L-Glutamyl-L-phenylalanine | 5.9E+02 | 2.6E+01 | 1.1E+03 | 7.2E+02 | 1.889 | 0.502 |
| Succinic acid | 9.2E+02 | 1.5E+02 | 7.8E+02 | 1.4E+02 | 0.841 | 0.517 |
| 3-Methyluridine | 2.0E+03 | 2.4E+02 | 1.6E+03 | 4.9E+02 | 0.812 | 0.528 |
| L-Glutamine | 5.1E+06 | 1.6E+05 | 5.6E+06 | 6.9E+05 | 1.095 | 0.531 |
| L-Dihydroorotic acid | 1.2E+03 | 2.5E+02 | 9.4E+02 | 3.7E+02 | 0.757 | 0.536 |
| Phosphorylcholine | 4.6E+04 | 2.4E+04 | 2.8E+04 | 1.3E+04 | 0.604 | 0.540 |
| 2'-O-methyladenosine | 1.4E+03 | 2.8E+02 | 1.6E+03 | 1.7E+02 | 1.152 | 0.557 |
| L-Carnitine | 5.0E+06 | 4.6E+05 | 5.5E+06 | 5.7E+05 | 1.090 | 0.572 |
| Adenine | 3.7E+04 | 4.2E+03 | 3.4E+04 | 4.5E+03 | 0.904 | 0.593 |
| Deoxycytidine | 1.1E+03 | 3.1E+02 | 1.5E+03 | 4.7E+02 | 1.287 | 0.595 |
| L-Aspartic Acid | 3.3E+04 | 2.7E+03 | 3.1E+04 | 6.8E+02 | 0.951 | 0.604 |
| cis-Aconitic acid | 5.6E+04 | 7.1E+03 | 5.0E+04 | 8.6E+03 | 0.892 | 0.614 |
| Creatinine | 9.0E+07 | 1.3E+06 | 9.1E+07 | 1.5E+06 | 1.011 | 0.652 |
| L-Citrulline | 2.2E+06 | 6.8E+04 | 2.3E+06 | 2.6E+05 | 1.060 | 0.653 |
| Glycyl-L-leucine | 1.8E+03 | 1.0E+02 | 2.1E+03 | 5.2E+02 | 1.130 | 0.671 |
| 5-hydroxy-Tryptophan | 9.2E+02 | 6.5E+01 | 8.3E+02 | 2.1E+02 | 0.898 | 0.687 |
| cis-4-Hydroxy-D-proline | 2.5E+05 | 8.3E+04 | 2.1E+05 | 5.3E+04 | 0.834 | 0.694 |
| gamma-Aminobutyric acid (GABA) | 3.0E+03 | 2.0E+02 | 3.1E+03 | 3.1E+02 | 1.050 | 0.706 |
| N2,N2-Dimethylguanosine | 5.3E+04 | 9.6E+03 | 5.8E+04 | 5.2E+03 | 1.083 | 0.707 |
| Cytosine | 7.6E+03 | 1.3E+03 | 8.5E+03 | 1.8E+03 | 1.119 | 0.707 |
| L-Pipecolic acid | 7.9E+04 | 1.6E+04 | 9.0E+04 | 2.5E+04 | 1.141 | 0.722 |
| Sarcosine | 5.3E+04 | 8.7E+03 | 5.9E+04 | 1.6E+04 | 1.128 | 0.729 |
| Salicyluric acid | 1.2E+03 | 2.3E+02 | 1.5E+03 | 9.3E+02 | 1.254 | 0.763 |
| myo-Inositol | 4.0E+03 | 2.7E+02 | 3.7E+03 | 8.3E+02 | 0.932 | 0.773 |
| Choline | 2.8E+05 | 1.1E+04 | 2.7E+05 | 2.3E+04 | 0.972 | 0.775 |
| Phenyllactic acid | 6.2E+03 | 6.0E+02 | 7.0E+03 | 2.8E+03 | 1.135 | 0.781 |
| Ornithine | 2.9E+06 | 3.3E+05 | 3.1E+06 | 9.4E+05 | 1.085 | 0.820 |
| N6-methyladenosine | 1.2E+05 | 2.4E+04 | 1.2E+05 | 1.5E+04 | 1.052 | 0.841 |
| L-Carnosine | 2.9E+03 | 1.4E+02 | 3.0E+03 | 4.7E+02 | 1.036 | 0.842 |
| Ethanolamine | 7.6E+03 | 8.5E+02 | 7.8E+03 | 6.3E+02 | 1.028 | 0.852 |
| NG,NG-Dimethyl-L-arginine (ADMA) | 1.3E+06 | 5.1E+04 | 1.2E+06 | 3.4E+05 | 0.946 | 0.852 |
| Uridine | 4.7E+03 | 2.6E+02 | 4.6E+03 | 2.1E+02 | 0.986 | 0.855 |
| S-Methyl-L-cysteine | 9.7E+03 | 2.8E+03 | 1.0E+04 | 2.2E+03 | 1.071 | 0.858 |
| Betaine | 1.2E+07 | 3.2E+06 | 1.1E+07 | 1.3E+06 | 0.950 | 0.869 |
| L-Kynurenine | 1.2E+05 | 1.4E+04 | 1.2E+05 | 6.8E+03 | 1.021 | 0.881 |
| N-Acetylcadaverine | 3.5E+04 | 4.7E+03 | 3.8E+04 | 1.8E+04 | 1.083 | 0.884 |
| N-Acetyl-L-alanine | 1.3E+04 | 2.0E+03 | 1.2E+04 | 1.6E+03 | 0.971 | 0.891 |
| Fumaric acid | 1.7E+03 | 1.8E+02 | 1.7E+03 | 1.0E+02 | 1.014 | 0.913 |
| DL-2-Aminooctanoic acid | 4.3E+04 | 1.1E+04 | 4.2E+04 | 1.2E+04 | 0.969 | 0.940 |
| L-Proline | 9.2E+06 | 2.5E+06 | 9.0E+06 | 3.2E+06 | 0.972 | 0.953 |
| Purine | 7.0E+03 | 1.5E+03 | 6.9E+03 | 9.6E+02 | 0.992 | 0.977 |
| L-Homoserine | 5.9E+05 | 2.7E+05 | 5.9E+05 | 1.1E+05 | 0.992 | 0.988 |
| Cytidine | 2.2E+04 | 3.0E+03 | 2.2E+04 | 2.5E+03 | 1.001 | 0.997 |
